# *cis*-Dichloridobis(dimethoxy­phenyl­phosphine)palladium(II)

**DOI:** 10.1107/S1600536810006471

**Published:** 2010-02-24

**Authors:** Alexandra M. Z. Slawin, Paul G. Waddell, J. Derek Woollins

**Affiliations:** a Department of Chemistry, University of St Andrews, St Andrews KY16 9ST, Scotland

## Abstract

The title compound, [PdCl_2_(C_8_H_11_O_2_P)_2_], has a comparable structure to those of related palladium dichloride complexes containing trimethyl phosphinite and methyl diphenyl phosphinite. The Pd atom is located on a crystallographic twofold rotation axis: thus, there is just one half-mol­ecule in the asymmetric unit. The structure is isomorphous with the platinum analogue *cis*-[PtCl_2_{P(OMe)_2_Ph}_2_].

## Related literature

For related structures, see: Slawin *et al.* (2009 ▶, 2007 ▶); Powell & Jacobson (1980 ▶). For preparation of the title compound, see: Jenkins & Shaw (1966 ▶).
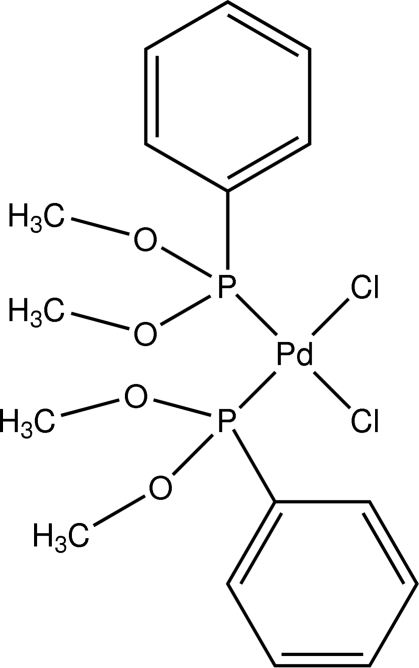


## Experimental

### 

#### Crystal data

[PdCl_2_(C_8_H_11_O_2_P)_2_]
*M* *_r_* = 517.60
Monoclinic, 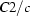

*a* = 10.876 (7) Å
*b* = 9.174 (5) Å
*c* = 20.722 (13) Å
β = 102.196 (7)°
*V* = 2021 (2) Å^3^
*Z* = 4
Mo *K*α radiation
μ = 1.36 mm^−1^
*T* = 125 K
0.16 × 0.15 × 0.09 mm

#### Data collection

Rigaku Mercury CCD diffractometer
Absorption correction: multi-scan (*ABSCOR*; Higashi, 1995 ▶) *T*_min_ = 0.812, *T*_max_ = 0.888
8406 measured reflections
1775 independent reflections
1653 reflections with *F* ^2^ > 2σ(*F* ^2^)
*R*_int_ = 0.032

#### Refinement

*R*[*F* ^2^ > 2σ(*F* ^2^)] = 0.023
*wR*(*F* ^2^) = 0.050
*S* = 1.09
1775 reflections
117 parameters
All H-atom parameters refined
Δρ_max_ = 1.15 e Å^−3^
Δρ_min_ = −0.32 e Å^−3^

### 

Data collection: *SCXmini* (Rigaku/MSC, 2006 ▶); cell refinement: *PROCESS-AUTO* (Rigaku, 1998 ▶); data reduction: *PROCESS-AUTO*; program(s) used to solve structure: *SHELXS97* (Sheldrick, 2008 ▶); program(s) used to refine structure: *SHELXL97* (Sheldrick, 2008 ▶); molecular graphics: *CrystalStructure* (Rigaku/MSC, 2006 ▶); software used to prepare material for publication: *CrystalStructure*.

## Supplementary Material

Crystal structure: contains datablocks global, I. DOI: 10.1107/S1600536810006471/bt5195sup1.cif

Structure factors: contains datablocks I. DOI: 10.1107/S1600536810006471/bt5195Isup2.hkl

Additional supplementary materials:  crystallographic information; 3D view; checkCIF report

## Figures and Tables

**Table:** 

Pd1—Cl1	2.3515 (16)
Pd1—P1	2.2300 (16)

**Table:** 

Cl1—Pd1—Cl1^i^	91.49 (2)
Cl1—Pd1—P1	174.86 (2)
Cl1—Pd1—P1^i^	83.85 (2)
P1—Pd1—P1^i^	100.88 (2)

Symmetry code: (i) 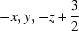.

**Fig. 1.**
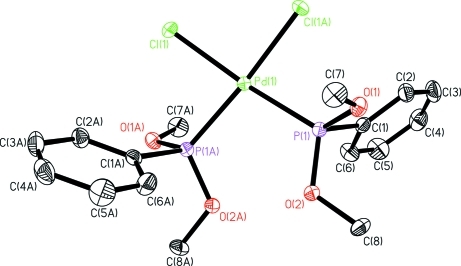
The structure of the title compound with displacement ellipsoids drawn at the 50% probability level, hydrogen atoms omitted for clarity. Symmetry operator for generating equivalent atoms (A): -x, y, 1.5-z.

## supplementary crystallographic information

### Experimental

Based on the method of Jenkins & Shaw (1966). To (0.3 g (0.93 mmol) of potassium tetrachloropalladate was dissolved in 10 ml e thanol and 0.147 ml (1.86 mmol) of P(OMe)_2_Ph was added. The solution was stirred at room temperature for 30 min s before being filtered and then precipitated by slow addition of hexane to give a white crystalline solid. Crystals were grown for X-ray crystallography via slow diffusion of hexane into a solution of the product in dichloromethane [cis-PdCl_2_(P(OMe)_2_Ph)_2_] (0.40 mmol, ca. 68 %). ^31^P-{^1^H} NMR: δ 125.8 ppm.

### Refinement

All H atoms were included in calculated positions (C—H distances are 0.96 Å for methyl H atoms, 0.93 Å for aromatic H atoms) and were refined as riding atoms with *U*_iso_(H) = 1.2 *U*_eq_(C) or *U*_iso_(H) = 1.5 *U*_eq_(C_methyl_). The highest peak in the difference map is 1.23 Å from atom Pd1.

### Crystal data

**Table:**

[PdCl_2_(C_8_H_11_O_2_P)_2_]	*F*(000) = 1040.00
*M**_r_* = 517.60	*D*_x_ = 1.701 Mg m^−^^3^
Monoclinic, *C*2/*c*	Mo *K*α radiation, λ = 0.71075 Å
Hall symbol: -C 2yc	Cell parameters from 2591 reflections
*a* = 10.876 (7) Å	θ = 2.9–25.4°
*b* = 9.174 (5) Å	µ = 1.36 mm^−^^1^
*c* = 20.722 (13) Å	*T* = 125 K
β = 102.196 (7)°	Prism, colorless
*V* = 2021 (2) Å^3^	0.16 × 0.15 × 0.09 mm
*Z* = 4	

### Data collection

**Table:**

Rigaku Mercury CCD diffractometer	1653 reflections with *F*^2^ > 2σ(*F*^2^)
Detector resolution: 0.83 pixels mm^-1^	*R*_int_ = 0.032
ω scans	θ_max_ = 25.0°
Absorption correction: multi-scan (*ABSCOR*; Higashi, 1995)	*h* = −13→13
*T*_min_ = 0.812, *T*_max_ = 0.888	*k* = −11→11
8406 measured reflections	*l* = −24→24
1775 independent reflections	

### Refinement

**Table:**

Refinement on *F*^2^	All H-atom parameters refined
*R*[*F*^2^ > 2σ(*F*^2^)] = 0.023	*w* = 1/[σ^2^(*F*_o_^2^) + (0.0162*P*)^2^ + 4.843*P*] where *P* = (*F*_o_^2^ + 2*F*_c_^2^)/3
*wR*(*F*^2^) = 0.050	(Δ/σ)_max_ = 0.001
*S* = 1.09	Δρ_max_ = 1.15 e Å^−^^3^
1775 reflections	Δρ_min_ = −0.32 e Å^−^^3^
117 parameters	

### Special details

**Table:**

Geometry. ENTER SPECIAL DETAILS OF THE MOLECULAR GEOMETRY
Refinement. Refinement was performed using all reflections. The weighted *R*-factor (wR) and goodness of fit (*S*) are based on *F*^2^. *R*-factor (gt) are based on *F*. The threshold expression of *F*^2^ > 2.0 σ(*F*^2^) is used only for calculating *R*-factor (gt).

### Fractional atomic coordinates and isotropic or equivalent isotropic displacement parameters (Å^2^)

**Table:**

	*x*	*y*	*z*	*U*_iso_*/*U*_eq_	
Pd1	0.0000	0.09671 (3)	0.7500	0.01397 (8)	
Cl1	−0.09211 (6)	−0.08216 (6)	0.67365 (3)	0.02090 (14)	
P1	0.10038 (6)	0.25152 (7)	0.82618 (3)	0.01521 (14)	
O1	0.24303 (15)	0.21038 (18)	0.85487 (8)	0.0189 (3)	
O2	0.10272 (16)	0.41579 (17)	0.80284 (8)	0.0199 (3)	
C1	0.0398 (2)	0.2542 (2)	0.90033 (12)	0.0172 (5)	
C2	0.0988 (2)	0.1823 (2)	0.95758 (12)	0.0220 (5)	
C3	0.0459 (2)	0.1856 (3)	1.01266 (13)	0.0285 (6)	
C4	−0.0648 (2)	0.2601 (3)	1.01080 (13)	0.0302 (6)	
C5	−0.1248 (2)	0.3298 (3)	0.95385 (14)	0.0328 (6)	
C6	−0.0733 (2)	0.3264 (2)	0.89844 (13)	0.0259 (5)	
C7	0.3242 (2)	0.1770 (3)	0.80984 (14)	0.0278 (6)	
C8	0.1786 (2)	0.5209 (2)	0.84682 (13)	0.0274 (6)	
H1	0.2993	0.0858	0.7883	0.033*	
H2	0.1736	0.1321	0.9588	0.026*	
H3	0.0851	0.1376	1.0510	0.034*	
H4	−0.0994	0.2634	1.0482	0.036*	
H5	−0.1999	0.3791	0.9528	0.039*	
H6	−0.1143	0.3724	0.8599	0.031*	
H7	0.4097	0.1704	0.8340	0.033*	
H8	0.3175	0.2528	0.7773	0.033*	
H9	0.1697	0.5038	0.8913	0.033*	
H10	0.1508	0.6179	0.8337	0.033*	
H11	0.2654	0.5103	0.8444	0.033*	

### Atomic displacement parameters (Å^2^)

**Table:**

	*U*^11^	*U*^22^	*U*^33^	*U*^12^	*U*^13^	*U*^23^
Pd1	0.01670 (15)	0.01215 (14)	0.01241 (14)	0.0000	0.00162 (10)	0.0000
Cl1	0.0256 (3)	0.0154 (3)	0.0190 (3)	−0.0012 (2)	−0.0014 (2)	−0.0041 (2)
P1	0.0170 (3)	0.0143 (3)	0.0137 (3)	−0.0012 (2)	0.0020 (2)	0.0000 (2)
O1	0.0154 (8)	0.0217 (8)	0.0194 (8)	−0.0021 (7)	0.0029 (6)	−0.0027 (7)
O2	0.0266 (9)	0.0151 (8)	0.0163 (8)	−0.0040 (6)	0.0002 (7)	0.0006 (6)
C1	0.0198 (12)	0.0159 (12)	0.0159 (11)	−0.0031 (9)	0.0037 (9)	−0.0022 (9)
C2	0.0212 (13)	0.0248 (14)	0.0188 (12)	0.0032 (10)	0.0017 (10)	−0.0012 (10)
C3	0.0336 (15)	0.0356 (16)	0.0153 (13)	−0.0020 (12)	0.0025 (11)	0.0020 (11)
C4	0.0363 (16)	0.0370 (16)	0.0203 (14)	−0.0054 (12)	0.0125 (12)	−0.0051 (12)
C5	0.0279 (15)	0.0383 (17)	0.0352 (16)	0.0073 (12)	0.0132 (12)	0.0003 (13)
C6	0.0265 (14)	0.0271 (14)	0.0245 (14)	0.0048 (11)	0.0062 (11)	0.0048 (11)
C7	0.0239 (14)	0.0317 (15)	0.0308 (15)	−0.0024 (11)	0.0127 (11)	−0.0017 (12)
C8	0.0387 (16)	0.0172 (13)	0.0249 (14)	−0.0079 (11)	0.0034 (12)	−0.0043 (11)

### Geometric parameters (Å, °)

**Table:**

Pd1—Cl1	2.3515 (16)	C4—C5	1.379 (3)
Pd1—Cl1^i^	2.3515 (16)	C5—C6	1.380 (4)
Pd1—P1	2.2300 (16)	C2—H2	0.930
Pd1—P1^i^	2.2300 (16)	C3—H3	0.930
P1—O1	1.5853 (16)	C4—H4	0.930
P1—O2	1.5846 (17)	C5—H5	0.930
P1—C1	1.795 (2)	C6—H6	0.930
O1—C7	1.446 (3)	C7—H1	0.960
O2—C8	1.458 (2)	C7—H7	0.960
C1—C2	1.390 (3)	C7—H8	0.960
C1—C6	1.391 (3)	C8—H9	0.960
C2—C3	1.383 (4)	C8—H10	0.960
C3—C4	1.378 (4)	C8—H11	0.960
			
O1···C3^ii^	3.324 (2)	H3···H9^ii^	2.979
O2···C7^iii^	3.551 (3)	H3···H11^ii^	2.774
C3···O1^ii^	3.324 (2)	H4···Cl1^xiv^	3.073
C3···C3^iv^	3.557 (3)	H4···C5^xii^	3.112
C7···O2^v^	3.551 (3)	H4···C8^xiii^	3.191
C7···C8^v^	3.542 (3)	H4···H4^xii^	3.467
C8···C7^iii^	3.542 (3)	H4···H5^xii^	2.542
Cl1···H3^vi^	3.536	H4···H7^ii^	2.908
Cl1···H4^vi^	3.073	H4···H9^xiii^	2.669
Cl1···H5^vii^	3.099	H4···H10^xiii^	2.840
Cl1···H6^vii^	3.149	H5···Cl1^xv^	3.099
Cl1···H7^v^	3.041	H5···C2^xi^	3.554
Cl1···H8^viii^	2.961	H5···C4^xii^	3.093
Cl1···H8^v^	3.311	H5···C5^xii^	3.552
Cl1···H10^ix^	2.822	H5···H2^xi^	2.715
O1···H3^ii^	2.775	H5···H4^xii^	2.542
O1···H6^x^	3.459	H5···H5^xii^	3.396
O2···H1^iii^	2.826	H5···H9^xiii^	3.354
O2···H7^xi^	3.294	H5···H10^viii^	3.575
C2···H2^ii^	3.196	H6···Cl1^xv^	3.149
C2···H3^iv^	3.535	H6···O1^xi^	3.459
C2···H5^x^	3.554	H6···C7^xi^	3.007
C3···H2^ii^	3.419	H6···H1^xi^	2.515
C3···H3^iv^	3.431	H6···H7^xi^	2.809
C3···H7^ii^	3.381	H6···H10^viii^	3.418
C4···H5^xii^	3.093	H6···H11^viii^	3.560
C4···H7^ii^	3.363	H7···Cl1^iii^	3.041
C4···H9^xiii^	3.329	H7···O2^x^	3.294
C5···H2^xi^	3.551	H7···C3^ii^	3.381
C5···H4^xii^	3.112	H7···C4^ii^	3.363
C5···H5^xii^	3.552	H7···C6^x^	3.417
C6···H1^xi^	3.384	H7···C8^x^	3.190
C6···H7^xi^	3.417	H7···H3^ii^	2.955
C6···H10^viii^	3.572	H7···H4^ii^	2.908
C6···H11^viii^	3.451	H7···H6^x^	2.809
C7···H3^ii^	3.313	H7···H9^x^	3.213
C7···H6^x^	3.007	H7···H10^x^	2.668
C7···H10^x^	3.523	H7···H10^v^	3.431
C7···H10^v^	3.092	H8···Cl1^xvi^	2.961
C7···H11^v^	3.491	H8···Cl1^iii^	3.311
C8···H1^iii^	2.922	H8···C8^v^	3.345
C8···H3^ii^	3.301	H8···H1^iii^	3.474
C8···H4^xiii^	3.191	H8···H10^v^	2.698
C8···H7^xi^	3.190	H8···H11^v^	3.341
C8···H8^iii^	3.345	H9···C4^xiii^	3.329
H1···O2^v^	2.826	H9···H2^ii^	3.445
H1···C6^x^	3.384	H9···H3^ii^	2.979
H1···C8^v^	2.922	H9···H4^xiii^	2.669
H1···H6^x^	2.515	H9···H5^xiii^	3.354
H1···H8^v^	3.474	H9···H7^xi^	3.213
H1···H10^v^	2.712	H10···Cl1^xvii^	2.822
H1···H11^v^	2.779	H10···C6^xvi^	3.572
H2···C2^ii^	3.196	H10···C7^xi^	3.523
H2···C3^ii^	3.419	H10···C7^iii^	3.092
H2···C5^x^	3.551	H10···H1^iii^	2.712
H2···H2^ii^	3.026	H10···H4^xiii^	2.840
H2···H3^ii^	3.409	H10···H5^xvi^	3.575
H2···H5^x^	2.715	H10···H6^xvi^	3.418
H2···H9^ii^	3.445	H10···H7^xi^	2.668
H3···Cl1^xiv^	3.536	H10···H7^iii^	3.431
H3···O1^ii^	2.775	H10···H8^iii^	2.698
H3···C2^iv^	3.535	H11···C6^xvi^	3.451
H3···C3^iv^	3.431	H11···C7^iii^	3.491
H3···C7^ii^	3.313	H11···H1^iii^	2.779
H3···C8^ii^	3.301	H11···H3^ii^	2.774
H3···H2^ii^	3.409	H11···H6^xvi^	3.560
H3···H3^iv^	3.552	H11···H8^iii^	3.341
H3···H7^ii^	2.955		
			
Cl1—Pd1—Cl1^i^	91.49 (2)	C1—C2—H2	120.1
Cl1—Pd1—P1	174.86 (2)	C3—C2—H2	120.1
Cl1—Pd1—P1^i^	83.85 (2)	C2—C3—H3	120.0
Cl1^i^—Pd1—P1	83.85 (2)	C4—C3—H3	120.0
Cl1^i^—Pd1—P1^i^	174.86 (2)	C3—C4—H4	119.8
P1—Pd1—P1^i^	100.88 (2)	C5—C4—H4	119.8
Pd1—P1—O1	114.07 (6)	C4—C5—H5	120.0
Pd1—P1—O2	115.26 (6)	C6—C5—H5	120.0
Pd1—P1—C1	113.66 (7)	C1—C6—H6	120.0
O1—P1—O2	105.27 (9)	C5—C6—H6	120.0
O1—P1—C1	100.91 (10)	O1—C7—H1	109.5
O2—P1—C1	106.30 (10)	O1—C7—H7	109.5
P1—O1—C7	119.39 (14)	O1—C7—H8	109.5
P1—O2—C8	118.82 (14)	H1—C7—H7	109.5
P1—C1—C2	122.50 (19)	H1—C7—H8	109.5
P1—C1—C6	117.70 (18)	H7—C7—H8	109.5
C2—C1—C6	119.7 (2)	O2—C8—H9	109.5
C1—C2—C3	119.8 (2)	O2—C8—H10	109.5
C2—C3—C4	120.1 (2)	O2—C8—H11	109.5
C3—C4—C5	120.4 (2)	H9—C8—H10	109.5
C4—C5—C6	120.0 (2)	H9—C8—H11	109.5
C1—C6—C5	119.9 (2)	H10—C8—H11	109.5
			
Cl1—Pd1—P1^i^—O1^i^	47.02 (7)	O1—P1—O2—C8	−46.3 (2)
Cl1—Pd1—P1^i^—O2^i^	169.01 (8)	O2—P1—O1—C7	−77.89 (18)
Cl1—Pd1—P1^i^—C1^i^	−67.93 (8)	O1—P1—C1—C2	−21.0 (2)
Cl1^i^—Pd1—P1—O1	47.02 (7)	O1—P1—C1—C6	161.81 (18)
Cl1^i^—Pd1—P1—O2	169.01 (8)	C1—P1—O1—C7	171.69 (17)
Cl1^i^—Pd1—P1—C1	−67.93 (8)	O2—P1—C1—C2	−130.59 (19)
P1—Pd1—P1^i^—O1^i^	−135.01 (7)	O2—P1—C1—C6	52.2 (2)
P1—Pd1—P1^i^—O2^i^	−13.03 (8)	C1—P1—O2—C8	60.2 (2)
P1—Pd1—P1^i^—C1^i^	110.03 (8)	P1—C1—C2—C3	−178.50 (19)
P1^i^—Pd1—P1—O1	−135.01 (7)	P1—C1—C6—C5	179.1 (2)
P1^i^—Pd1—P1—O2	−13.03 (8)	C2—C1—C6—C5	1.8 (3)
P1^i^—Pd1—P1—C1	110.03 (8)	C6—C1—C2—C3	−1.3 (3)
Pd1—P1—O1—C7	49.44 (18)	C1—C2—C3—C4	−0.1 (3)
Pd1—P1—O2—C8	−172.89 (16)	C2—C3—C4—C5	1.0 (4)
Pd1—P1—C1—C2	101.57 (19)	C3—C4—C5—C6	−0.6 (4)
Pd1—P1—C1—C6	−75.6 (2)	C4—C5—C6—C1	−0.8 (4)

Symmetry codes: (i) −*x*, *y*, −*z*+3/2; (ii) −*x*+1/2, −*y*+1/2, −*z*+2; (iii) −*x*+1/2, *y*+1/2, −*z*+3/2; (iv) −*x*, −*y*, −*z*+2; (v) −*x*+1/2, *y*−1/2, −*z*+3/2; (vi) *x*, −*y*, *z*−1/2; (vii) −*x*−1/2, *y*−1/2, −*z*+3/2; (viii) *x*−1/2, *y*−1/2, *z*; (ix) −*x*, *y*−1, −*z*+3/2; (x) *x*+1/2, *y*−1/2, *z*; (xi) *x*−1/2, *y*+1/2, *z*; (xii) −*x*−1/2, −*y*+1/2, −*z*+2; (xiii) −*x*, −*y*+1, −*z*+2; (xiv) *x*, −*y*, *z*+1/2; (xv) −*x*−1/2, *y*+1/2, −*z*+3/2; (xvi) *x*+1/2, *y*+1/2, *z*; (xvii) −*x*, *y*+1, −*z*+3/2.
